# CHEK2 signaling is the key regulator of oocyte survival after chemotherapy

**DOI:** 10.1126/sciadv.adg0898

**Published:** 2023-10-20

**Authors:** Chihiro Emori, Zachary Boucher, Ewelina Bolcun-Filas

**Affiliations:** ^1^ The Jackson Laboratory, 600 Main Street, Bar Harbor, ME 04609, USA.; ^2^ Department of Experimental Genome Research, Research Institute for Microbial Diseases, Osaka University, Suita, Osaka 5650871, Japan.

## Abstract

Cancer treatments can damage the ovarian follicle reserve, leading to primary ovarian insufficiency and infertility among survivors. Checkpoint kinase 2 (CHEK2) deficiency prevents elimination of oocytes in primordial follicles in female mice exposed to radiation and preserves their ovarian function and fertility. Here, we demonstrate that CHEK2 also coordinates the elimination of oocytes after exposure to standard-of-care chemotherapy drugs. CHEK2 activates two downstream targets—TAp63 and p53—which direct oocyte elimination. CHEK2 knockout or pharmacological inhibition preserved ovarian follicle reserve after radiation and chemotherapy. However, the lack of specificity for CHEK2 among available inhibitors limits their potential for clinical development. These findings demonstrate that CHEK2 is a master regulator of the ovarian cellular response to damage caused by radiation and chemotherapy and warrant the development of selective inhibitors specific to CHEK2 as a potential avenue for ovario-protective treatments.

## Teaser

Specific inhibition of CHEK2 during cancer treatment is a potential strategy to preserve ovarian function in cancer patients.

## INTRODUCTION

Women are born with a finite supply of primordial follicles (PMFs) that constitute the ovarian reserve. Hormonal sufficiency and optimal female health depend on a robust supply of follicles in the ovary. This is because developing follicles are a major source of female hormones such as estrogens that are responsible for the development and regulation of the female reproductive system and overall reproductive health. Estrogen is also important for bone and cardiovascular health. Activated PMFs grow and ultimately produce the eggs that are ovulated; therefore, they are necessary for female fertility. PMFs decrease naturally with age until menopause, but studies in human and animal models show that various environmental factors—such as pollutants, smoking, diet, medical treatments, etc.—can diminish the ovarian reserve (*1*). The ovarian reserve can also be damaged by cancer treatments leading to accelerated PMF loss and primary ovarian insufficiency (POI) in cancer survivors (*2*). Treatment-induced POI results in early menopause, a premature endocrine deficiency associated with increased risk of osteoporosis, cardiovascular disorders, depression, and other comorbidities that require medical attention and can negatively affect the quality of survivorship (*3*). Quality-of-life considerations are especially important for pediatric, adolescent, and young adult cancer survivors due to their long-life expectancy. The overall prevalence of POI varies considerably due to heterogeneity of cancers and treatments (*4*–*6*). Fertility preservation methods, such as cryopreservation of eggs and embryos, are well established and available for cancer patients of reproductive age (*7*, *8*). For prepubertal girls, ovarian tissue cryopreservation is the only fertility preservation option for systemic cancer treatments (*9*). Thawed ovarian cortical tissue auto-transplantation later in life successfully restored fertility and prolonged ovarian endocrine function in cancer survivors (*10*, *11*). However, there are currently no pharmacological and nonsurgical methods to preserve follicle reserve and long-term ovarian endocrine function after chemotherapy-induced damage (*9*, *12*). Drug treatments preserving ovarian follicles without surgical removal of whole ovaries may be favored by patients who do not wish to have children, or have already cryopreserved their eggs or embryos. Studies in mice, primates, and human ovarian xenografts demonstrated the potential of Sphingosine-1-phosphate and its mimetic FTY720 as protective agents against radiation- and chemotherapy-induced POI and infertility (*13*–*16*).

Genotoxic cancer treatments kill cancer cells by inducing DNA damage in the form of single-strand breaks (SSBs) and double-strand breaks (DSBs), which are more detrimental to fast-dividing cancer cells than healthy cells. However, these treatments can also damage healthy cells including oocytes. DNA damage inflicted in primary oocytes residing in PMFs (henceforth “primordial oocytes”) is the major trigger of radiation- and chemotherapy-induced PMF elimination (*17*). In contrast, primary oocytes in growing follicles (primary, secondary, or preantral follicles; henceforth “growing oocytes”) are more resistant to DNA damage–induced apoptosis, although the underlying mechanism remains largely unknown (*18*). Therefore, blocking the DNA damage response (DDR) that triggers apoptosis in primordial oocytes, thus allowing more time for DNA repair, offers an attractive strategy to prevent PMF loss and POI. We showed that genetic inactivation of checkpoint kinase 2 (CHEK2) prevents radiation-induced PMF loss in mice (*19*). Moreover, CHEK2-deficient females exposed to radiation gave birth to normal numbers of healthy offspring indicating that oocytes that survived radiation can repair DNA damage. In somatic cells, DDR signaling triggers cell cycle arrest to allow DNA repair before apoptosis is triggered. In contrast, oocytes in the ovary are naturally arrested at meiotic prophase I for an extended period of time. It could take months or years before arrested primordial oocytes resume meiosis before ovulation, which would provide sufficient time to repair DNA damage. However, owing to the strong meiotic DNA damage checkpoint coordinated by CHEK2, primordial oocytes are eliminated before they can repair DNA damage. Therefore, temporarily blocking the CHEK2-dependent response could provide more time to repair DNA damage and avoid triggering apoptosis in oocytes, thus protecting PMFs from elimination.

The most lethal type of DNA damage—DSBs—leads to activation of CHEK2. In contrast, SSBs predominantly activate CHEK1 (*20*). CHEK2 coordinates DDR through activation of TRP53 (henceforth p53), which leads to cell cycle arrest, senescence, or apoptosis depending on the cell type and cell cycle phase (*21*). However, in primordial oocytes, CHEK2 predominantly activates a p53-related protein TRP63—specifically its TA isoform (henceforth TAp63)—leading to oocyte elimination (*19*, *22*). Genetic inactivation of TAp63 has been reported to protect primordial oocytes from cisplatin (CDDP)–induced damage but not from cyclophosphamide (CTX) (*23*, *24*). These differences in survival suggest involvement of other DDR proteins (i.e., CHEK1 and p53) in primordial oocytes in response to different chemotherapy drugs. Studies utilizing kinase inhibitors known to target both CHEK1 and CHEK2 reported their protective effects in primordial oocytes against CDDP, CTX, and doxorubicin (DOX) toxicity (*22*, *24*, *25*). However, the limited selectivity of these inhibitors obfuscates which of the CHEK kinases are activated by which chemotherapy drug. Considering the potential use of CHEK2 inhibitors for primordial oocyte protection, more information is needed regarding CHEK2 activation and its downstream effectors in chemotherapy-induced POI to identify cancer treatments that could benefit from oocyte-protective activity of CHEK2 inhibitors.

Here, we use wild-type and CHEK2-deficient mice to determine the role of CHEK2 in oocyte elimination in response to chemotherapy-induced damage and whether inactivation of CHEK2 or its downstream effectors is sufficient to prevent chemotherapy-induced loss of PMFs. We provide genetic evidence that CHEK2 inhibition is sufficient to protect primordial oocytes from alkylating agents CDDP, CTX, and mafosfamide (MAFO), and topoisomerase II poisons DOX and etoposide (ETO). We also present genetic evidence that both TAp63 and p53 are involved in elimination of primordial oocytes damaged by alkylating agents CDDP and MAFO. To probe therapeutic avenues, we show that the cancer-sensitizing agent and CHEK1/2 dual inhibitor AZD7762 can prevent radiation- and chemotherapy-induced primordial oocyte elimination. However, doses of AZD7762 needed for oocyte protection were cytotoxic to ovarian somatic cells due to potent inhibition of CHEK1. Three other inhibitors tested—CCT241533, LY2606368, and PF477736—showed no protective effect in an ex vivo system. The results of this study highlight the importance of targeted development of selective inhibitors specific to CHEK2 as potential treatments preventing PMF loss during genotoxic cancer therapy.

## RESULTS

### Genetic inactivation of CHEK2 prevents oocyte elimination caused by chemotherapy drugs in ex vivo organ culture

**Fig. 1.**
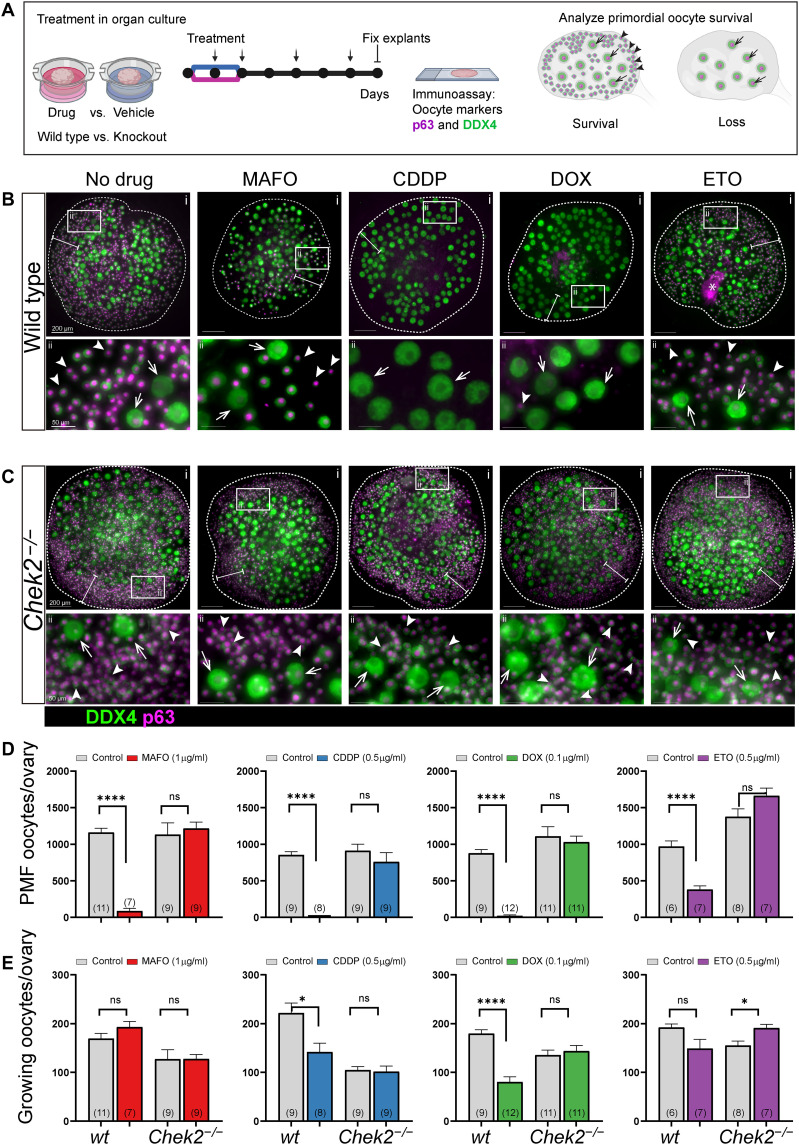
CHEK2 deficiency prevents primordial oocyte depletion after ex vivo treatment with MAFO, CDDP, DOX, and ETO. (**A**) Schematic representation of the ovary explant organ culture system. Explants were exposed to drugs for 48 hours, then monitored in drug-free culture for 5 days (7 days total, circles = days). Media changes are indicated by arrows. Whole ovaries from wild-type (**B**) and *Chek2^−/−^* (**C**) females after 7-day ex vivo culture with MAFO (1 μg/ml), CDDP (0.5 μg/ml), DOX (0.1 μg/ml), or ETO (0.5 μg/ml) were immunostained for oocyte markers DDX4 (cytoplasmic green) and p63 (nuclear magenta). Representative whole ovaries are shown in the top row (i) and white boxes mark regions magnified below (ii). White bars indicate regions where primordial follicles (PMFs) are typically found in cultured ovaries. Small cells marked by arrowheads in magnified regions indicate primordial oocytes in PMF (sensitive to treatments) and large cells marked by arrows indicate oocytes in growing follicles (resistant to treatments). Asterisk: unspecific staining. Scale bars, 200 μm for whole ovary images and 50 μm for magnified regions. (**D**) Primordial and (**E**) growing oocytes were counted in ovaries harvested after 7-day organ culture. (*N*) = number of ovaries per group. Data are expressed as mean ± SEM; *P < 0.05, ****P < 0.0001 (Mann-Whitney nonparametric test). (A) was created with BioRender.

Previous studies showed that genetic inactivation of CHEK2 prevents POI in mice after exposure to radiation (*19*,*26*). Studies using the CHEK inhibitor BML-277 showed a protective effect against the chemotherapy drugs CDDP, DOX, and CTX derivative 4-HC (*22*, *24*, *25*). However, it remains unclear whether this protective effect is due to specific inhibition of CHEK2, CHEK1, or both kinases due to limited selectivity. To test the direct role of CHEK2 in the elimination of PMFs damaged by genotoxic chemotherapy drugs and determine whether CHEK2 inhibition would be effective and sufficient in reducing PMF loss, we tested whether genetic ablation of CHEK2 function prevents primordial oocyte elimination after treatments. We chose chemotherapy drugs known to induce DNA damage via different mechanisms and characterized by different severities of ovarian adverse effects: DNA alkylating agents CDDP and MAFO, and DNA topoisomerase II poisons DOX and ETO (*27*). We used MAFO, a preactivated CTX derivative that can be used in ex vivo culture to imitate the in vivo activity of CTX, which needs to be metabolized by the liver to activate its alkylating properties. However, both drugs have been shown to generate similar metabolites (*28*). To better control for effective drug doses and duration of exposure for comparative analysis, we utilized an ex vivo organ culture system (Fig. 1A), where we cultured whole ovaries to prevent potential PMF activation due to fragmentation of ovarian tissue (*29*). We used ovaries from 1-week-old females, the stage at which mouse ovaries predominantly contain fully formed PMFs and a small population of primary/secondary follicles. This approach also facilitates investigation of direct toxicity in primordial oocytes without the potential confounding effects from damage to large growing follicles seen in postpubertal ovaries. Ovaries were cultured for 5 days after completion of 48 hours of drug treatment, or a total of 1 week, to ensure observation of long-term primordial oocyte survival rather than delayed apoptosis. To determine doses that deplete primordial oocytes in mice, we exposed ovarian explants from wild-type females to increasing doses of MAFO (0.1 to 1 μg/ml), CDDP (0.1 to 0.5 μg/ml), DOX (0.025 to 0.1 μg/ml), and ETO (0.05 to 0.5 μg/ml) (fig. S1). Doses used in this ex vivo study are lower than the reported plasma concentrations in patients at the highest single recommended dose [CDDP: 4.3 μg/ml, CTX: 33.4 μg/ml, DOX: 3.66 μg/ml, and ETO: 19.66 μg/ml (*30*, *31*)], yet they are sufficient to significantly deplete primordial oocytes in mice. PMFs lack direct contact with blood vessels; drugs reach the oocytes through diffusion, which may explain the toxicity at lower doses in organ culture. The number of primordial oocytes was reduced compared to vehicle controls after exposure to all tested drugs in a dose-dependent manner (fig. S1). Significantly reduced survival of primordial oocytes was observed in wild-type ovaries for MAFO at 1 μg/ml (7.3% ± 9.0%; *P* < 0.0001), CDDP at 0.5 μg/ml (0.04% ± 0.08%; *P* < 0.0001), and DOX at 0.1 μg/ml (2.4% ± 0.6%; *P* < 0.0001), and to lesser extent for ETO at 0.5 μg/ml (39.0% ± 13.7%; *P* = 0.0021) (Fig. 1, B and D). In agreement with published data, ETO showed lowest toxicity in juvenile ovaries, suggesting mechanistic differences in activity compared to the other topoisomerase II poison tested (DOX). When *Chek2^−/−^* ovaries were treated with the same doses of drugs, no significant reduction in primordial oocyte numbers was observed (Fig. 1, C and D). We calculated oocyte survival as a percentage of ovarian reserve in untreated wild-type and *Chek2^−/−^* females. When compared to wild-type controls, survival of primordial oocytes lacking CHEK2 was improved after MAFO (7.3% versus 107.3% ± 23.7%; *P* = 0.0002), CDDP (0.04% versus 83.2% ± 42.32%; *P* < 0.0001), DOX (2.4% versus 92.9% ± 24%; *P* < 0.0001), and ETO (39% versus 120.7% ± 20.3%; *P* = 0.0006).

Because there have been reports that CDDP and CTX induce hyperactivation of PMFs and their transition to growing follicles (*32*–*34*), we counted the larger growing oocytes (diameter > 30 μm), which are typically found in primary and secondary follicles, in 2-week-old ovaries treated with each of the four drugs. We observed no significant increase in growing oocyte numbers: To the contrary, in some cases (CDDP and DOX), we observed a reduction in the number of growing oocytes after drug treatments (Fig. 1E and fig. S1). This indicates that follicle hyperactivation is not the major mechanism of PMF loss in prepubertal ovaries or in ex vivo explants and is in agreement with other studies (*25*, *35*, *36*). In summary, survival of primordial oocytes in *Chek2^−/−^* ex vivo–treated ovaries demonstrates that CHEK2 is directly responsible for coordinating elimination of PMFs after treatment with four different chemotherapy drugs and that CHEK2 inhibition would likely be sufficient to prevent loss of PMF reserve in cancer patients treated with these drugs.

### CHEK2 deficiency prevents PMF loss after in vivo treatment with an acute dose of alkylating chemotherapy drugs

**Fig. 2.**
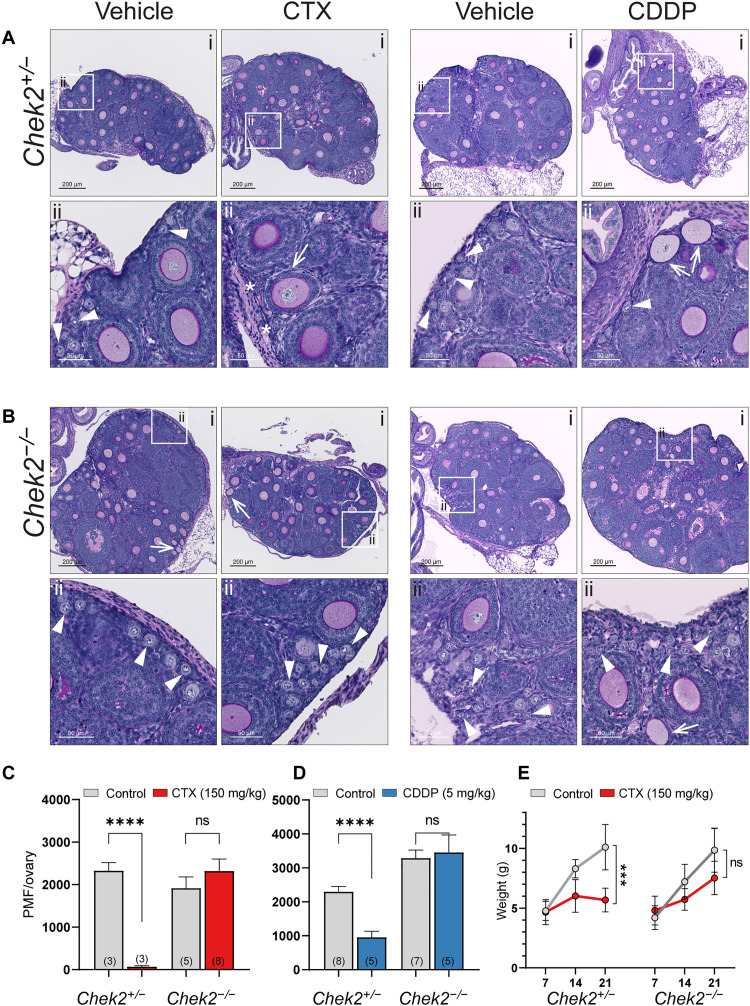
CHEK2-deficient females maintain PMF reserve after in vivo treatment with CTX and CDDP. Ovarian histology from *Chek2^+/−^* (**A**) and *Chek2^−/−^* (**B**) females 2 weeks after in vivo treatment with CTX (150 mg/kg) and CDDP (5 mg/kg). Representative sections are shown in the top row (i) and white boxes mark regions magnified below (ii). Arrowheads indicate PMFs and asterisks indicate follicle remnants devoid of oocytes. Arrows indicate growing follicles with abnormal granulosa cell layers. Abnormal growing follicles are occasionally found in untreated 3-week-old ovaries. Scale bars, 200 μm for whole ovary sections and 50 μm for magnified regions. (**C** and **D**) PMF numbers counted in *Chek2^+/−^* and *Chek2^−/−^* ovaries 2 weeks after injection with CTX and CDDP, respectively. Data are expressed as mean ± SEM; ****P < 0.0001, ns, nonsignificant (Mann-Whitney nonparametric test). (**E**) Body weight changes in *Chek2^+/−^* and *Chek2^−/−^* females injected with CTX (7 to 21 dpp). (*N*) = number of females per group. Data are expressed as mean ± SEM; ***P < 0.001, ns, nonsignificant (Mann-Whitney nonparametric test).

To confirm that abrogation of CHEK2 activity is sufficient to mitigate chemotherapy-induced PMF loss in prepubertal mice treated in vivo with highly ovotoxic alkylating agents CTX and CDDP (*37*), 1-week-old control (*Chek2^+/−^*) and *Chek2^−/−^* females were treated with vehicle or an acute dose of CTX (150 mg/kg) or CDDP (5 mg/kg). Female weights were recorded before treatment and then weekly after. Ovaries were collected 2 weeks after treatment (at 3 weeks of age) for histological analyses of the PMF reserve. CTX treatment obliterated oocytes in PMFs in control females where follicle remnants were often observed (3.1% ± 2.2%; *P* < 0.0001) (Fig. 2, A and C). In contrast, abundant PMFs with oocytes were present in ovaries from CTX-treated *Chek2^−/−^* females (91% ± 40.8% versus 3.1%; *P* < 0.0001) (Fig. 2, B and C). CTX treatment caused additional side effects in pups including hair loss and growth retardation. Improved weight gain was evident in treated *Chek2^−/−^* pups compared to controls (60% ± 14.3% versus 10% ± 7.2%) (Fig. 2E), suggesting that inhibition of CHEK2 activity could potentially alleviate other adverse side effects caused by CTX, although hair loss was not prevented. CDDP treatment significantly decreased PMF numbers in ovaries from control females (38.7% ± 26.5%; *P* = 0.0002), but to a lesser extent than CTX (Fig. 2, A and D). Compared to treated controls, *Chek2^−/−^* females showed abundant PMFs (126% ± 59.6% versus 38.7%; *P* = 0.0009) as well as growing follicles (Fig. 2, B and D). Overall, CTX- and CDDP-treated *Chek2^−/−^* females retained >90% of their ovarian reserve and showed healthy primary and secondary follicles in the ovary at 3 weeks of age and thus were expected to be fertile. Here, we focused on PMF survival rather than fertility outcomes, but other studies indicate that oocytes that survive genotoxic treatments can produce healthy offspring (*23*, *24*). Together, these results indicate that CHEK2 is predominantly if not exclusively responsible for triggering primordial oocyte elimination and that targeting CHEK2 activity in vivo will be sufficient to protect PMFs against CDDP- and CTX-induced damage.

### CDDP and MAFO treatments cause abundant DNA DSBs in oocytes, which exhibit markers for homologous recombination but not nonhomologous end joining repair

**Fig. 3.**
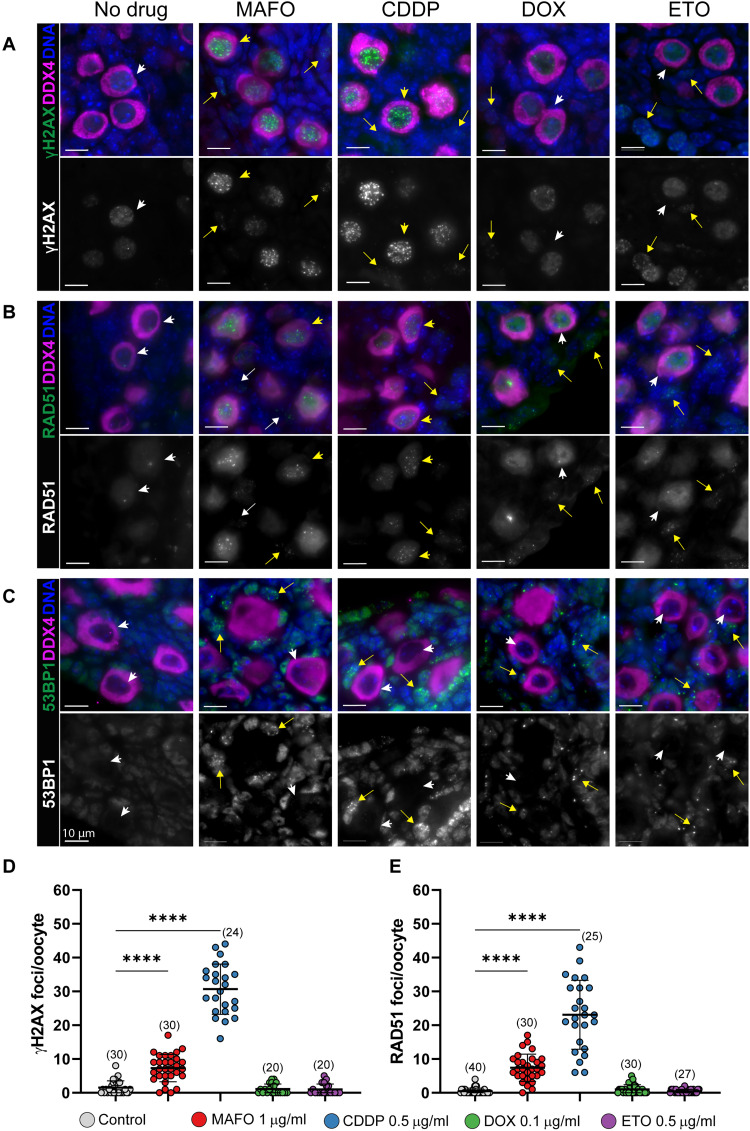
MAFO and CDDP treatment of oocytes causes DNA DSBs that activate HR, but not NHEJ, repair. Ovaries were exposed to vehicle and chemotherapy drugs ex vivo for 24 hours (MAFO: 1 μg/ml, CDDP: 0.5 μg/ml, DOX: 0.1 μg/ml, and ETO 0.5 μg/ml). Oocytes in PMF were analyzed by immunostaining for general DNA damage marker γ-H2AX (**A**), DNA repair markers RAD51 for HR (**B**), and 53BP1 for NHEJ (**C**) in green as indicated on the left. Background levels of γ-H2AX and RAD51 are detected in untreated primordial oocytes (white arrowheads). Oocytes were labeled with DDX4 (magenta) and DNA counterstained with Hoechst (blue). Top panels show merged representative immunofluorescence images and bottom panels show corresponding grayscale image of DNA damage and repair markers. Arrowheads and arrows indicate primordial oocytes and somatic cells, respectively. Yellow arrowheads and arrows show cells with DNA DSBs and white arrowheads and arrows show cells without damage. Scale bars, 10 μm. (**D** and **E**) Quantification of the number of DSBs per oocyte using γ-H2AX (D) and RAD51 (E) markers. Sample number (*N*); number of cells per group. Data are expressed as mean ± SD; ****P < 0.0001 (one-way ANOVA, Bonferroni multiple-comparison test).

CHEK2 has been shown to coordinate elimination of oocytes in response to unrepaired, programmed meiotic and radiation-induced DSBs (*19*). To examine whether the four tested drugs cause DNA DSBs in oocytes, which would activate CHEK2 signaling, we immunostained vehicle- and drug-treated wild-type ovaries for general DNA DSB marker γ-H2AX. After 24 hours of treatment, abundant γ-H2AX foci were present in all primordial oocytes in ovaries treated with MAFO and CDDP. However, in ovaries treated with DOX or ETO, elevated levels of γ-H2AX foci were rarely observed in oocytes (Fig. 3, A and D). DNA damage markers were present in granulosa and other ovarian cell types after all four treatments, indicating that drug doses used in this study induce DNA damage (Fig. 3A). Because DSBs can be repaired by high-fidelity homologous recombination (HR) or more error-prone nonhomologous end joining (NHEJ), we immunostained treated ovaries for HR marker RAD51 and NHEJ marker 53BP1.

RAD51 foci were present in oocytes treated with MAFO or CDDP, indicating that these drugs activate the HR repair pathway (Fig. 3, B and E). RAD51 foci were rarely observed in DOX- and ETO-treated oocytes, consistent with the γ-H2AX staining results, while RAD51 foci were found in somatic cells (Fig. 3, B and E). Oocytes in postnatal ovaries are arrested at dictyate stage of meiotic prophase I, comparable to mitotic G2 phase. In this phase, HR is the predominant repair pathway due to the presence of sister chromatids, but NHEJ repair is also thought to be active. A previous report shows that DNA-PKcs, a component of NHEJ repair, is detected in 10% of primordial oocytes after radiation-induced DNA damage (*38*). Another study reported DNA-PKcs activation in 30 to 60% of oocytes after CTX treatment (*39*). 53BP1 directs DSB repair toward NHEJ and localizes to DSB sites forming discrete foci that colocalize with γ-H2AX (*40*). Remarkably, there was no evidence for 53BP1 foci in oocytes treated with any of our tested chemotherapy drugs (Fig. 3C). However, they were readily detected in somatic cells in treated ovaries (Fig. 3C). This indicates that DNA damage in oocytes is predominantly, if not exclusively, repaired by high-fidelity HR, which has a lower risk of generating deleterious mutations in surviving oocytes, as was shown for radiation (*38*). Lack of DNA damage markers in primordial oocytes within 24 hours of DOX and ETO treatments suggests that DSBs may not be the primary type of damage caused by these drugs that leads to CHEK2 activation and oocyte apoptosis. It is possible that DSB formation after DOX treatment is delayed or caused indirectly by oxidative stress as γ-H2AX–positive primordial oocytes were previously reported in human and mouse ovaries after DOX treatment (*41*). Additionally, differences in accumulation of DNA damage in oocytes could be due to interspecies differences in drug pharmacokinetics and metabolism.

**Fig. 4.**
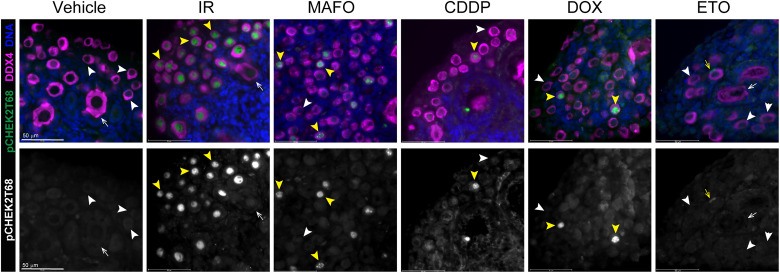
Radiation and chemotherapy treatments lead to activation of CHEK2 in oocytes. Ovaries were exposed to radiation and chemotherapy drugs ex vivo for 24 hours (IR: 0.5 Gy, MAFO: 1 μg/ml, CDDP: 0.5 μg/ml, DOX: 0.1 μg/ml, and ETO: 0.5 μg/ml). Ovarian sections were immunostained for phospho-CHEK2 T68 (green) and DDX4 (magenta) as indicated on the left. pCHEK2 T68 is readily detected in all primordial oocytes exposed to radiation. Few pCHEK2-positive oocytes are also detected in MAFO-, CDDP-, and DOX-treated ovaries, while rare pCHEK2-positive granulosa cells are detected on ETO-treated ovaries (yellow arrowheads and yellow arrows). Scale bars, 50 μm. Top panels show merged image and bottom panels show corresponding grayscale image of pCHEK2.

To determine whether CHEK2 is activated in oocytes by chemotherapy drugs, we immunostained drug-treated ovaries for CHEK2 phosphorylated at threonine-68 (*42*). Of note, we found that multiple commercially available antibodies either do not detect CHEK2 in mouse paraffin-fixed ovaries by standard methods or showed unspecific staining also present in CHEK2-deficient samples. However, we identified one specific antibody against pCHEK2 T68 that shows expected nuclear signal in wild-type but not in *Chek2^−/−^* ovaries treated with radiation. Activated pCHEK2 was readily detected in primordial oocytes 3 hours after radiation and was still present in oocytes 24 hours after radiation (Fig. 4). MAFO, CDDP, and DOX treatment showed delayed CHEK2 activation compared to radiation; pCHEK2 was not detected in primordial oocytes treated for 3 to 6 hours. Primordial oocytes positive for pCHEK2 were present in ovaries treated for 24 hours, but not as abundant as those damaged by radiation. CHEK2 activation was rarely detected in ETO-treated oocytes. This delayed and asynchronous pattern of CHEK2 activation most likely reflects a more complex mechanism of drug toxicity for alkylating agents compared to instant physical damage caused by radiation. Why CHEK2 activation, measured as ATM-dependent T68 phosphorylation (*43*), is not readily detected in all primordial oocytes after treatment with MAFO and CDDP—although they are positive for DNA damage markers (Fig. 3, A and B)—remains unclear and will need additional studies or better reagents. Nevertheless, our genetic data clearly demonstrate that CHEK2 is involved in elimination of primordial oocytes after chemotherapy treatments.

Together, we show that CDDP and MAFO induce lethal DSBs in prophase I–arrested primordial oocytes and in dividing ovarian somatic cells. Repair of DSBs in oocytes is initiated by high-fidelity HR but cannot be completed due to activation of proapoptotic responses coordinated by CHEK2. Moreover, our results confirm previous reports that topoisomerase II poisons such as DOX and ETO do not induce abundant DSBs in oocytes at least within 24 hours of treatment (*44*), but rather in ovarian somatic cells that are actively proliferating. Nevertheless, primordial oocyte elimination is still observed in CHEK2-proficient ovaries and increased survival in CHEK2-deficient ovaries, indicating that DOX and ETO affect primordial oocyte survival by a different mechanism, potentially related to oxidative stress. Additional studies are needed to identify how cellular damage caused by these drugs leads to CHEK2-dependent oocyte elimination. In summary, our results indicate that inhibition of CHEK2 activity in chemotherapy-treated ovary prevents elimination of primordial oocytes with and without DNA DSBs by providing more time for repair of cellular damage and allowing oocyte survival.

### Damage induced by CDDP and MAFO does not induce hyperphosphorylation of TAp63 and triggers activation of p53-dependent apoptosis

**Fig. 5.**
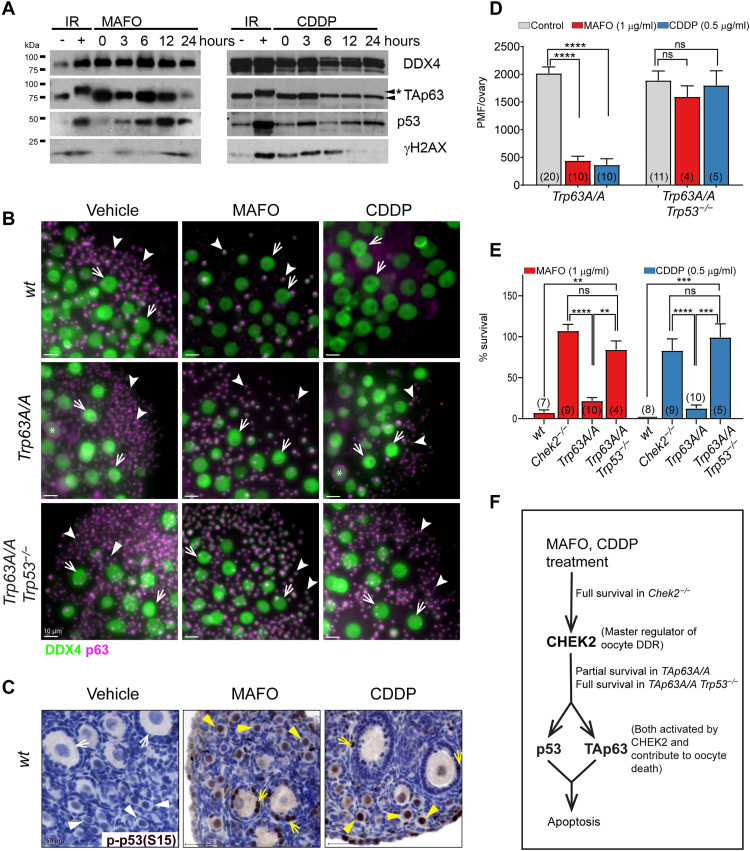
Inhibition of CHEK2-dependent TAp63 phosphorylation is not sufficient to prevent complete primordial oocyte elimination in response to MAFO (1 μg/ml) and CDDP (0.5 μg/ml) treatments indicating a role for p53. (**A**) CDDP and MAFO fail to induce TAp63 hyperphosphorylation within 24 hours after treatment. TAp63 hyperphosphorylation leads to mobility shift (asterisk) observed after radiation. Compared to untreated controls, increased levels of total p53, indicative of its phosphorylation and stabilization, are observed after drug treatments. DDX4 (oocyte marker); γ-H2AX (DNA damage marker). (**B**) Primordial oocytes expressing nonphosphorylatable mutant TAp63 are not fully resistant to MAFO and CDDP toxicity while double mutant oocytes lacking active TAp63 and p53 are highly resistant and display almost normal survival. Ovaries from wild-type, *Trp63A/A* (mutation at S621A), and *Trp63A/A Trp53^−/−^* double mutant females after 7-day ex vivo culture after treatments with MAFO and CDDP were immunostained for oocyte markers DDX4 (green) and p63 (magenta). Representative immunofluorescence images of ovaries are shown. Scale bars, 50 μm. Arrowheads indicate oocytes in PMF and arrows indicate oocytes in growing follicles. Asterisk: oocytes with abnormal localization of p63 staining. (**C**) phospho-p53 detection in oocytes and granulosa cells after treatment with MAFO and CDDP by IHC. (**D**) Primordial oocyte (PMF) counts in ovaries treated with MAFO and CDDP. (**E**) Survival rates (%) were normalized to the average PMF number of the control group in each genotype (wild type, *Chek2^−/−^*, *Trp63A/A*, and *Trp63A/A Trp53^−/−^*). Sample number (*N*); number of ovaries per group. Data are expressed as mean ± SEM; **P < 0.01, ***P < 0.001, ****P < 0.0001 (Mann-Whitney nonparametric test). (**F**) Primordial oocyte survival in mouse mutants used in this study reveals the contribution of p53 and TAp63 to oocyte apoptosis.

In response to radiation-induced damage, CHEK2 phosphorylates two proapoptotic factors: TAp63 specifically in oocytes and p53 in all cell types (*19*, *22*, *45*). Unlike p53, which requires phosphorylation to avoid degradation (*45*), TAp63 is constitutively expressed in primordial oocytes and maintained in an inactive form until phosphorylated by CHEK2 (*19*, *22*). Phosphorylated TAp63 is detected in irradiated ovaries as mobility shift on Western blots (Fig. 5A and fig. S2C) (*22*, *46*). Reports suggest that inactivation of TAp63 is sufficient to protect primordial oocytes from CDDP-induced damage (*23*, *24*), but may not be sufficient for CTX as two studies report contradictory results (*23*, *47*). Because MAFO and CDDP induce DSBs and trigger CHEK2-dependent apoptosis, phosphorylation and activation of TAp63 are expected. However, TAp63 shift was not detected up to 24 hours of MAFO or CDDP treatment, suggesting that majority of primordial oocytes failed to activate TAp63 in this timeframe (Fig. 5A). These results suggest that damage induced by MAFO (2.4 μM) and CDDP (1.6 μM) does not lead to rapid phosphorylation of TAp63 as observed after radiation where instant physical damage induces DDR. Activation of CHEK2 by CDDP and MAFO was also delayed compared to radiation; therefore, it is possible that DNA damage caused by alkylating agents triggers activation of CHEK2 and subsequently TAp63 with different dynamics (Fig. 4). This is supported by reports showing TAp63 phosphorylation after ex vivo treatments with a higher dose of CDDP (10 μM) (*22*, *24*) or after CTX treatment in vivo (*39*, *47*).

To determine whether TAp63 is involved in MAFO- and CDDP-induced primordial oocyte elimination downstream of CHEK2, we utilized a novel mouse model with a mutation in the *Trp63* gene (fig. S2A). Here, serine 621 was replaced with alanine (S621>A, S582>A in human) at the TAp63 C terminus, which was previously shown to be phosphorylated by CHEK2 (*19*, *22*). This mutation prevents activation by CHEK2 but does not affect TAp63 expression (fig. S2, C and D) and results in increased resistance of oocytes to low dose of radiation (0.5 Gy) (fig. S2D). *TAp63A/A* mutant females are fertile with normal ovarian histology (fig. S2B). We exposed *TAp63A/A* mutant ovarian explants ex vivo to MAFO and CDDP at the same doses and regimen as wild-type and *Chek2^−/−^* ovaries (Fig. 5, B and E, and Fig. 1). After treatment with MAFO and CDDP, primordial oocyte numbers were significantly reduced in *TAp63A/A* ovaries (Fig. 5, B and D). Primordial oocyte survival after MAFO treatment was lower in *TAp63A/A* ovaries at *~*21.9% ± 12.6% (*P* < 0.0001) versus ~107.3% in *Chek2^−/−^* (*P* < 0.0001), and after CDDP treatment at ~12.7% ± 13% (*P* < 0.0001) versus ~83.2% (*P* < 0.0001) (Fig. 5E). The reduced number of surviving primordial oocytes in *TAp63A/A* mutants suggests that other CHEK2 target/s contribute to primordial oocyte elimination after MAFO- and CDDP-induced damage. We detected phospho-p53(S15) in treated ovaries and specifically in primordial oocytes by immunostaining (Fig. 5C). Phospho-p53 was also detected in primordial oocytes after treatment with CTX in vivo (*39*). To test whether p53 contributes to primordial oocyte elimination after MAFO- and CDDP-induced damage, we analyzed oocyte survival in double mutant ovaries lacking activity of both TAp63 and p53 (*48*). Compared to *Trp63A/A* single mutant, primordial oocyte survival was significantly improved in *Trp63A/A Trp53^−/−^* double mutant ovaries after MAFO (84.4% ± 21% versus 21.9%; *P* = 0.002) and CDDP treatment (99.4% ± 36.4% versus 12.7%; *P* = 0.001) (Fig. 5, B and E). This confirms that p53 is activated in oocytes after damage with alkylating agents and that it contributes to primordial oocyte elimination. CDDP induces higher number of DSBs than MAFO in ovarian explants (Fig. 3D); therefore, it is possible that p53 activation in oocytes depends on the amount of cellular damage induced by chemotherapy drugs. Together, genetic analysis reveals the critical role for CHEK2 in coordinating PMF elimination after MAFO- and CDDP-induced damage by activating two proapoptotic factors TAp63 and p53 (Fig. 5F). Moreover, loss of CHEK2 or p53 in ovarian somatic cells is expected to prevent apoptosis in ovarian somatic cells and thus indirectly contribute to primordial oocyte survival.

### Transient pharmacological inhibition of CHEK2 improves primordial oocyte survival after radiation, CDDP, and MAFO treatments

**Fig. 6.**
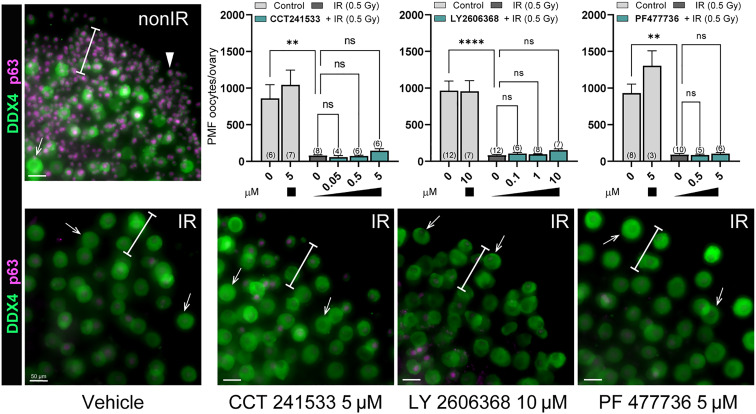
CCT241533, LY2606368 and PF477736 fail to prevent primordial oocyte elimination after radiation treatment. Ovaries were treated with inhibitors ex vivo for 2 hours before IR and for 24 hours after IR. After 24 hours, inhibitors were withdrawn, and ovaries were cultured for six additional days without inhibitors. Graphs show numbers of oocytes per ovary present after treatment with increasing doses of inhibitors (μM). Sample number (*N*); number of ovaries per group. Data are expressed as mean ± SEM; **P < 0.01, ****P < 0.0001 (one-way ANOVA, Kruskal-Wallis with Dunn’s multiple-comparison test for nonparametric data). Below, panels show examples of treated ovaries immunostained with oocyte markers DDX4 (green) and p63 (magenta). White bars indicate regions where PMFs are typically found in cultured ovaries. Arrowheads indicate primordial oocytes and arrows denote larger growing oocytes. Scale bar, 50 μm.

We showed that genetic ablation of CHEK2 activity prevents depletion of PMF reserve in mice treated with chemotherapy drugs (this study) and radiation (*19*). Because of similarities in structure and function between CHEK1 and CHEK2, many available checkpoint kinase inhibitors have limited selectivity and can block the activity of both kinases, albeit with different affinities (*49*). CHEK1 is an essential kinase that coordinates DDR and cell cycle progression in all dividing cells and shares many downstream targets with CHEK2 (*50*). CHEK1/2 inhibitors have been shown to potentiate the effects of genotoxic chemotherapy drugs against cancer and some are being tested in clinical trials (*51*–*53*). Therefore, inhibitors blocking CHEK2 and CHEK1 could have dual benefits for female patients: improved cancer cell elimination and oocyte protection. To test the principle that CHEK1/2 inhibitors could prevent oocyte depletion after radiation and chemotherapy treatments, we selected four inhibitors shown or predicted to target CHEK2 and to some degree CHEK1, and tested them in ovarian explant culture: AZD7762 (*54*), CCT241533 (*55*), LY2606368 (*56*), and PF477736 (*57*). The pharmacokinetics of these inhibitors in mouse is unknown. Therefore, we used our ex vivo ovarian explant culture system to better control the timing and dosing of drugs and inhibitors without the confounding influence of transport and metabolism found in vivo. We treated ovarian explants with increasing doses of inhibitors in combination with low-dose ionizing radiation 0.5 Gy known to deplete primordial oocytes as described previously (*26*). Ovarian explants were precultured with inhibitors for 2 hours before radiation to assure cellular presence of the inhibitor before DNA damage and for additional 24 hours after radiation. Primordial oocyte numbers were counted in immunostained whole ovaries after an additional six days of culture without inhibitor to ensure quantification of oocyte survival and not a delay in apoptosis. Treatment with CCT241533 (0.05, 0.5, and 5 μM), LY2606368 (0.1, 1, and 10 μM), and PF477736 (0.5 and 5 μμ) had no impact on primordial oocyte survival after radiation at any dose tested (Fig. 6), indicating failure to sufficiently block CHEK2 activity in oocytes although similar doses have been used in cell culture with cancer cell lines.

**Fig. 7.**
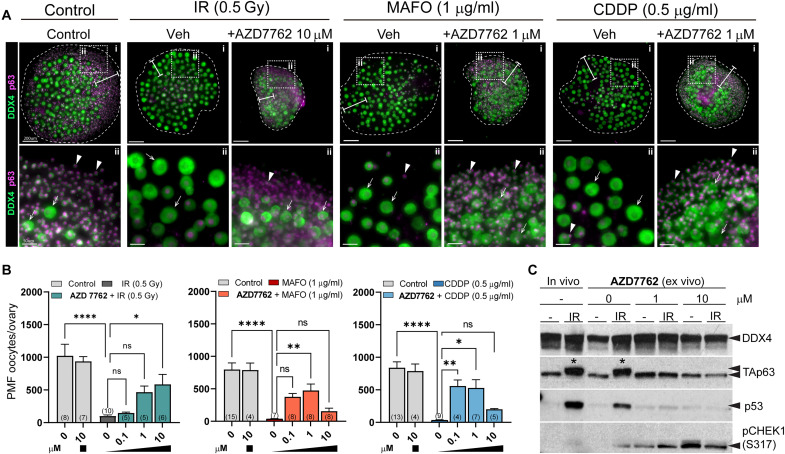
Cotreatment with AZD7762 reduces primordial oocyte loss in prepubertal ovaries after treatment with radiation and chemotherapy drugs. (**A**) Cotreatment with AZD7762 exhibited protective effect and presence of primordial oocytes after treatments. Example ovaries cotreated with AZD7762 and radiation, MAFO, or CDDP in ex vivo organ culture. Ovaries were treated with AZD7762 ex vivo for 2 hours before treatments and for 48 hours after treatment start. Inhibitors and drugs were withdrawn, and ovaries were cultured for five additional days without inhibitors or drugs. Oocyte markers DDX4 (green) and p63 (magenta). Representative whole ovaries are shown in the top row (i) and dotted boxes mark regions magnified below (ii). White bars indicate regions where primordial oocytes are typically found in cultured ovaries. Arrowheads indicate primordial oocytes and arrows denote larger growing oocytes. Scale bars, 200 μm for whole ovary images and 50 μm for magnified regions. (**B**) Graphs show numbers of primordial oocytes (PMF oocytes) per ovary present after cotreatment with increasing doses of AZD7762 inhibitor. Sample number (*N*); number of ovaries per group. Data are expressed as mean ± SEM; *P < 0.05, **P < 0.01, ****P < 0.0001 (one-way ANOVA, Kruskal-Wallis with Dunn’s multiple-comparison test for nonparametric data). (**C**) Ovarian extracts with and without AZD7762 treatment were analyzed by Western blot for activation of CHEK2 targets TAp63 and p53. Ovarian protein extracts were collected 6 hours after IR with 0.5 Gy in vivo or ex vivo. In contrast to ovaries irradiated without AZD7762, TAp63 mobility shift (asterisk) indicative of phosphorylation and p53 expression were not detected in AZD7762-treated ovaries. Increased levels of pCHEK1(S317) were present only in AZD7762-treated ovaries indicating accumulation of CHEK1.

In contrast, AZD7762 treatment improved primordial oocyte survival compared to vehicle-treated irradiated ovaries in a dose-dependent manner, although growth of the ovarian explants was retarded (Fig. 7A). We calculated survival as a percentage of oocyte reserve in nonirradiated ovaries. Survival was estimated at 57.3% ± 36.2% after 10 μM AZD7762 (*P* = 0.001) and 45.6% ± 20.6% (*P* = 0.008) after 1 μM AZD7762 versus 10.1% ± 4.4% with vehicle. To test whether AZD7762 could also protect primordial oocytes against chemotherapy drugs, ovaries were cotreated with AZD7762 (0.1, 1, and 10 μM) and MAFO or CDDP (Fig. 7A). More primordial oocytes survived chemotherapy-induced damage after cotreatment with AZD7762 (Fig. 7A); 59.6% ± 32.4% versus 4.5% ± 2.7% compared to MAFO alone (*P* = 0.0003) and 63.1% ± 42.5% versus 3.9% ± 2.4% (*P* = 0.0006) in CDDP alone. To validate that AZD7762 indeed prevents primordial oocyte death by inhibition of CHEK2-dependent signaling, we tested activation of TAp63 and p53 by Western blot. As expected, TAp63 was phosphorylated after radiation, which resulted in mobility shift and p53 was readily detected (Fig. 7C). In ovaries treated with AZD7762, TAp63 remained unphosphorylated and p53 was reduced (Fig. 7C), which confirms that AZD7762 efficiently inhibited CHEK2 activity in oocytes and prevented TAp63 activation and oocyte apoptosis. Because of lack of detectable TAp63 mobility shift after MAFO and CDDP treatments (Fig. 5A), activation of TAp63 was not tested for these drugs.

**Fig. 8.**
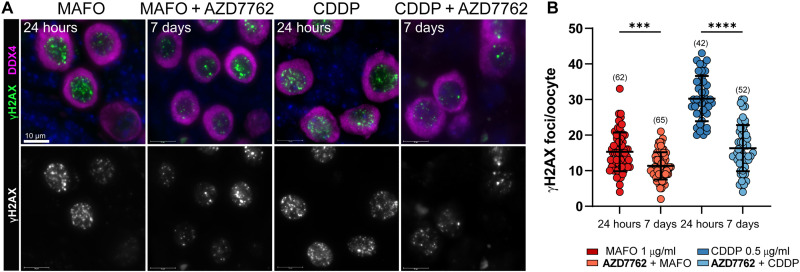
Inhibition of CHEK2 with AZD7762 facilitates DNA damage repair after treatment with chemotherapy drugs. (**A**) Drug-treated oocytes in PMF were analyzed by immunostaining for general DNA damage marker γ-H2AX after 24 hours treatment with MAFO (1 μg/ml) or CDDP (0.5 μg/ml) and after 48 hours cotreatment with AZD7762 (1 μM) at the end of 7 days of culture. Oocytes were labeled with DDX4 (magenta) and DNA was counterstained with Hoechst (blue). Top panels show merged representative immunofluorescence images and bottom panels show corresponding grayscale image of DNA damage marker. Scale bars, 10 μm. (**B**) Quantification of the number of γ-H2AX foci at 24 hours and 7 days of culture. (*N*); number of cells per group. Data are expressed as mean ± SD; ***P < 0.001, ****P < 0.0001 (one-way ANOVA, Bonferroni multiple-comparison test).

AZD7762 blocked activation of CHEK2 during drug treatment and improved oocyte survival. This suggests that inhibition of the CHEK2 pathway resulted in sufficient DNA repair to evade apoptosis even after inhibitor withdrawal. To determine whether AZD7762 inhibition allowed DSB repair, we analyzed γ-H2AX staining in ovaries 24 hours after treatment with MAFO or CDDP and 5 days after drug and inhibitor withdrawal (7 days total). Although γ-H2AX was still present in surviving primordial oocytes, the number of foci was significantly lower at the end of culture for MAFO 11.3 ± 3.8 versus 15.3 ± 5.4 (*P* = 0.0001) and CDDP 16.3 ± 6.4 versus 30.24 ± 6.4 (*P* < 0.0001) (Fig. 8). DSB repair assessment, beyond the time allowed by explant culture, may be needed to determine the full repair capacity of the surviving oocytes. Reduction in γ-H2AX foci suggests that transient inhibition of CHEK2-dependent checkpoint with AZD7762 prevents triggering apoptosis and enables DNA repair.

**Fig. 9.**
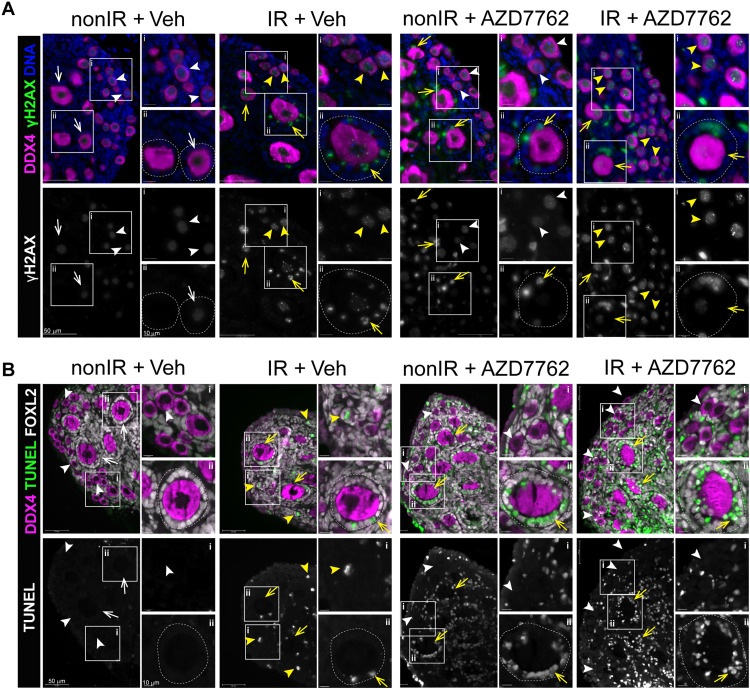
Dual CHEK1/2 inhibitor AZD7762 exhibits cytotoxic effects in proliferating ovarian somatic cells. Ovarian explants treated with vehicle or AZD7762 for a total of 8 hours without and with radiation (0.5 Gy) were immunostained for oocyte marker DDX4 (magenta), DNA damage marker γ-H2AX (green), apoptosis marker TUNEL (green), and granulosa cells marker FOXL2 (white). DAPI (blue). (**A**) AZD7762 treatment caused increased levels of γ-H2AX staining in somatic cells of growing follicles (arrows) but not oocytes even in the absence of IR. Background levels of γ-H2AX are detected in healthy oocytes (white arrowheads). Bright γ-H2AX foci indicate IR-induced DNA damage in primordial oocytes (yellow arrowheads) and granulosa cells in growing follicles (yellow arrows). Scale bars, 50 and 10 μm. Top panels show merged image and bottom panels show grayscale image. White boxes indicate magnified regions (i, ii). (**B**) TUNEL staining shows increased apoptosis in granulosa cells of growing follicles in ovaries treated with AZD7762. Yellow arrows show growing follicles with apoptotic granulosa cells. Scale bars, 10 and 50 μm. Growing follicles are outlined.

Despite improved oocyte survival, retardation of ovarian growth was also observed in cotreatment with chemotherapy drugs resulting in visibly smaller explants after 7 days of culture (Fig. 7A). This suggests that at doses required for CHEK2 inhibition and oocyte protection, AZD7762 strongly inhibited CHEK1 and impaired proliferation of ovarian somatic cells and ovarian growth. Accumulation of pCHEK1(S317) was detected by Western blot in all AZD7762-treated ovaries (Fig. 7C). CHEK1 is phosphorylated at serine-317 by ATR kinase in response to replication blocks and some forms of genotoxic stress (*58*). Immunostaining for DNA damage marker γ-H2AX and terminal deoxynucleotidyl transferase–mediated deoxyuridine triphosphate nick end labeling (TUNEL) for apoptosis showed that AZD7762 treatment alone caused accumulation of DNA damage (Fig. 9A) and increased apoptosis in ovarian somatic cells (Fig. 9B), particularly granulosa cells of growing follicles. AZD7762 treatment did not cause DNA damage in primordial oocytes; therefore, its effects appear to be restricted to proliferating cells. Because *Chek2^−/−^* ovaries show normal growth after drug treatment (Fig. 1), these results suggest that AZD7762 inhibited CHEK1 kinase that interfered with cell cycle progression in proliferating stromal and granulosa cells, which triggered cell death independently of CHEK1 and CHEK2 signaling. In summary, these data show that transient inhibition of CHEK2 is a feasible approach to protect ovarian PMF reserve during genotoxic chemotherapy treatments, providing that either less cytotoxic dual CHEK1/2 or highly selective CHEK2 inhibitor can be used.

## DISCUSSION

Genotoxic cancer treatments can damage and diminish PMF reserve in cancer patients leading to POI, thereby accelerating reproductive aging among cancer survivors. However, the safest and most effective way to mitigate PMF loss and reduce the risk of POI in female cancer patients remains unclear. Here, we show that CHEK2 kinase is responsible for coordinating elimination of primordial oocytes after damage with several chemotherapy drugs, making CHEK2 or its downstream effector proteins attractive targets for the development of ovario-protective treatments. Genetic ablation of CHEK2 protected >90% of PMF reserve in female mice treated in vivo with two highly ovotoxic drugs, CTX and CDDP. Although fertility was not assessed here, evidence from prior work and other studies indicate that primordial oocytes that survive genotoxic insults do indeed support normal ovarian function and fertility (*19*, *23*, *24*, *26*, *59*, *60*). Moreover, genetic inactivation of CHEK2 almost completely protected primordial oocytes from MAFO, CDDP, DOX, and ETO toxicity in the ex vivo organ culture system. Although DOX and ETO are not considered as ovotoxic as alkylating agents in human (*37*), they are typically used in combination therapies (*61*). Thus, our findings suggest that the protective effect of CHEK2 inhibition would likely be beneficial for a broad spectrum of patient treatments. Further, our genetic dissection of the signaling mechanisms responsible for PMF depletion in response to genotoxic insult establishes CHEK2 as a master regulator of this process and reveals parallel and redundant downstream pathways that involve both TAp63 and p53. Finally, we demonstrate that a dual CHEK1/2 inhibitor known to sensitize cancer cells to chemotherapy significantly improved primordial oocyte survival after treatment with radiation or alkylating agents ex vivo, suggesting that this class of cancer drugs could have an additional, beneficial effect against chemotherapy-induced ovarian toxicity.

How different chemotherapy drugs inflict damage in ovaries and in meiotically arrested oocytes specifically, and how this damage triggers primordial oocyte elimination is still not fully understood. Chemotherapy drugs can damage DNA both directly and indirectly by increasing oxidative stress (*62*, *63*). Here, we show that alkylating agents CDDP and MAFO (CTX analog) induce abundant DNA DSBs in oocytes [as previously reported (*64*)] that, in turn, trigger CHEK2-dependent oocyte apoptosis. In contrast, DSB markers were rarely found in primordial oocytes after DOX or ETO treatment, suggesting a different mode of toxicity leading to CHEK2 activation. It is possible that there are dose-dependent requirements and/or species-specific differences in DSB induction by DOX in mouse and human oocytes, as γ-H2AX positive oocytes have been reported in human ovaries cultured with higher doses of DOX (1 to 100 μg/ml) (*41*). DOX and ETO are known to induce cytotoxic oxidative damage in heart, kidney, and blood cells (*63*, *65*, *66*). It is possible that these drugs induce indirect DNA damage in oocytes as a result of elevated reactive oxygen species (ROS) that accumulate over time. Co-administration of drugs reducing oxidative stress has been shown to decrease their ovotoxicity (*65*–*67*). The improved survival of primordial oocytes in *Chek2^−/−^* ovaries after DOX and ETO exposure suggests that these drugs induce CHEK2 signaling independently of nuclear DNA damage, and likely through increased oxidative stress (*68*–*71*). A recent study reported complete primordial oocyte survival after DOX treatment in TAp63 oocyte-specific knockout, suggesting that CHEK2 triggers TAp63-dependent apoptosis in response to DOX-induced damage (*47*). Previous studies have suggested that the depletion of ovarian reserve caused by CDDP and CTX is due to the overactivation of PMFs and their transition into growing follicles, which are mediated by the activation of the PI3K/PTEN/AKT pathway (*32*–*34*). Here, we show that blocking the DDR through the inactivation of CHEK2/p53/TAp63 pathway prevents PMF depletion, evidence that DNA damage–induced apoptosis eliminates oocytes after CDDP and CTX treatment. This indicates that DNA damage–induced apoptosis plays a crucial role in the elimination of primordial oocytes after treatment with CDDP and CTX. Our results align with a transcriptomic study revealing activation of apoptotic pathways in human primordial oocytes treated with CTX. In contrast, the activity of the PI3K/PTEN/AKT pathway, indicative of follicle activation, was diminished (*36*). Another recent study found that overexpression of PI3K in primordial oocytes prevented CTX-induced apoptosis, suggesting that elevated levels of PI3K and activated follicle state could reduce oocyte sensitivity to DNA damage–induced apoptosis (*47*).

The primordial oocyte response to DNA DSBs induced by radiation involves CHEK2 and its downstream target TAp63, with p53 considered to be dispensable (*72*, *73*). Genetic and biochemical evidence indicate that hyperphosphorylation by CHEK2 is required to fully activate oocyte-specific proapoptotic factor TAp63 (*19*, *22*). However, the role of CHEK2 and TAp63 in the response to alkylating agents has been unclear due to reported discrepancies in TAp63 hyperphosphorylation status and its requirement for oocyte elimination. TAp63 hyperphosphorylation was not evident after CDDP and MAFO treatments in this study and (*24*, *74*) but has been reported after ex vivo treatment with a higher dose of CDDP (*22*, *24*) (10 μM versus 1.6 μM in this study) and in vivo administration of CTX (*39*, *47*). Differences in detection of TAp63 phosphorylation most likely reflect a dynamic response to DNA damage induced by different doses and modes of drug delivery such as diffusion in culture system versus blood circulation. While TAp63 null females are reported to be resistant to CDDP by two independent studies (*23*, *24*), their resistance to CTX remains unclear as two studies report conflicting results (*23*, *47*).

A study using an array of DDR inhibitors suggested that TAp63 can be activated by two distinct signaling cascades: ATM → CHEK2 → TAp63 for x-ray irradiation and ATR → CHEK1 → TAp63 for CDDP (*24*). Because many DDR inhibitors have limited selectivity and off-target effects (*75*, *76*) and there is a cross-talk between ATM and ATR pathways (*77*), it is possible that survival attributed to ATR → CHEK1 inhibition is due to off-target inhibition of the CHEK2 pathway. Using a genetic approach, we demonstrate here that CHEK2 is directly responsible for elimination of primordial oocytes damaged by both CDDP and CTX/MAFO and that CHEK1 activity does not substantially contribute to oocyte elimination even in the absence of CHEK2.

Radiation and alkylating agents are known to induce DSBs in oocytes and other cell types, but the mechanisms for the ensuing DDR and activation of apoptosis may not be shared. In contrast to the *TAp63* null genetic model (*23*), we observed that primordial oocytes expressing the phosphomutant TAp63—lacking the CHEK2 phosphorylation site associated with hyperphosphorylation—were sensitive to CDDP although CHEK2-deficient primordial oocytes were resistant to the same dose. This difference in survival raises two intriguing possibilities: First, CHEK2-dependent phosphorylation at different sites may contribute to TAp63 activation by CDDP without inducing a hyperphosphorylated state [as alluded by Kim *et al.* (*24*)]; second, other proapoptotic proteins regulated by CHEK2 are involved. Because TAp63 substitutes for p53 function during elimination of oocytes with unrepaired meiotic DSBs (*19*), we favor the latter explanation. We show that deleting *Trp53* in TAp63 phosphomutant background results in CDDP resistance, indicating that p53 can contribute to elimination of CDDP-damaged primordial oocytes. It is possible that TAp63 phosphomutant protein triggers oocyte apoptosis by forming an active heterotetramer with p53. Although heterotypic interactions have not been detected between wild-type p53 and TAp63 in somatic cells (*78*, *79*), further investigation is needed to exclude this possibility in oocytes.

**Fig. 10.**
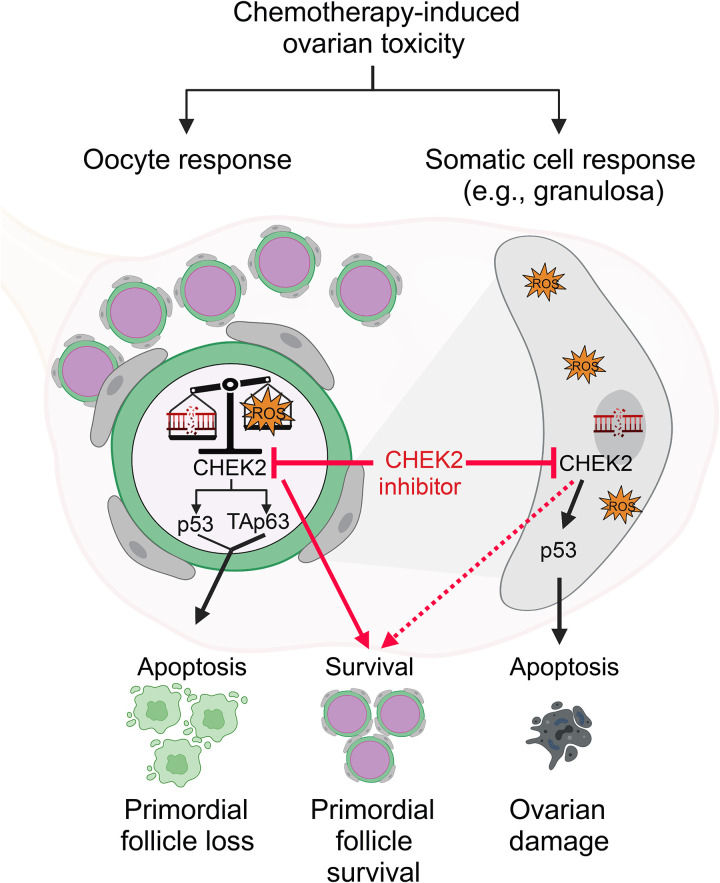
Schematic illustration of the conclusions of this study. Many chemotherapy drugs, including CDDP, CTX, DOX, and ETO, induce DNA damage and oxidative stress, which kill cancer cells (on-target effect), but they can also damage healthy cells such as those in the primordial follicles in ovaries, leading to their death (off-target effect). CHEK2 kinase coordinates response to chemotherapy toxicity by activating p53 in somatic cells, and p53 and/or TAp63 in oocytes depending on the amount and type of damage (e.g., DNA damage versus oxidative stress). Low levels of cellular damage are sufficient to activate TAp63-dependent apoptosis while p53 is activated at higher levels of DNA damage and oxidative stress; thus, the combined action of both pro-apoptotic factors regulates oocyte survival and death. CHEK2 inhibitors prevent oocyte elimination by blocking CHEK2 and its downstream signaling (red lines). CHEK2 and p53 are expressed in all cell types, therefore blocking CHEK2 activity in somatic cells in the ovary (e.g., granulosa) may indirectly contribute to primordial follicle survival (dashed line). Figure created with BioRender.com

We show that full survival of primordial oocytes in MAFO-treated ovaries was achieved only after both TAp63 and p53 were inactivated, suggesting that MAFO-induced damage activates both targets of CHEK2 independently or that they can substitute for each other. A previous study reported very low transcriptional activity for TAp63 after CTX treatment compared to p53, which showed high activity toward shared target *Bbc3* (*Puma*) (*39*). This suggests that p53 may be predominantly responsible for oocyte apoptosis after CTX-induced damage. However, two studies report conflicting results regarding PMF survival after CTX in TAp63-deficient females. Treatment of postpubertal TAp63 null females with CTX resulted in PMF elimination, supporting a role for p53 (*23*). In contrast, treatment of female pups with conditional deletion of TAp63 in oocytes resulted in PMF survival, suggesting that p53 is dispensable for oocyte elimination (*47*). Further investigation is needed to determine exactly what leads to activation of p53 versus TAp63-dependent apoptosis by different chemotherapy drugs. Clues to the regulation of TAp63 and p53 activation may emerge from comparisons of the cellular damage induced by different chemotherapy drugs and radiation. Low-dose radiation, which induces a small number of DSBs (*80*), seems to induce predominantly TAp63-dependent apoptosis (*72*, *73*). However, at higher doses of radiation, TAp63-deficient oocytes are eliminated, most likely by p53 (*80*). Radiation is known to induce dose-dependent increase in DSB numbers as well as oxidative stress (*81*), suggesting that either higher number of DSBs or oxidative stress activates TAp63-independent apoptosis. In addition to DSBs, CTX and its derivatives produce reactive metabolites, such as phosphoramide mustard and acrolein, that cause overproduction of ROS, which is known to activate the CHEK2 → p53 pathway (*71*, *82*). This suggests that MAFO/CTX activate CHEK2 and its downstream targets TAp63 and p53 by DNA damage–dependent and –independent mechanisms. Surprisingly, a recent report of PMF survival in DOX-treated TAp63-deficient females (*47*) suggests that DOX activates CHEK2 → TAp63–dependent apoptosis, despite the lack of direct DNA damage in oocytes (this study). However, these findings may also suggest that TAp63 responds primarily to oxidative stress and low levels of DSBs, while p53 responds to higher levels of DNA damage (*83*). CDDP and CTX/MAFO treatments induce high numbers of DSBs and oxidative stress, and therefore can activate both p53 and TAp63. Further studies, separating the effects of oxidative stress and DNA damage, are needed to dissect which type of damage activates TAp63 and p53 in oocytes. On the basis of this investigation and existing literature, we propose a new model of primordial oocyte response to chemotherapy: one suggesting that their survival depends on the type and amount of total cellular damage, and combined activity of p53 and TAp63 coordinated by master regulator CHEK2 (Fig. 10).

DNA damage inflicted in oocytes, either directly or indirectly, seems to be the major trigger of PMF elimination after radiation and chemotherapy; therefore, blocking the DDR signaling responsible for this elimination may be a promising strategy to prevent POI during cancer treatments. Inhibition of TAp63 and p53 protects primordial oocytes from low-dose radiation and selected chemotherapies (*24*, *47*, *72*, *73*, *80*), but targeting transcription factors remains challenging and blocking apoptosis directly may pose an issue for cancer treatment and development of resistance to therapy. Consequently, inhibition of DDR kinases, such as CHEK2, responsible for triggering oocyte elimination may be the best approach. Inhibitors targeting checkpoint kinases CHEK1/2 are being developed and tested as chemotherapy sensitizers (*84*), and they offer an attractive strategy to treat cancer and limit drug toxicity in ovaries. We show here that cotreatment with dual CHEK1/2 inhibitor AZD7762 can minimize chemotherapy-induced loss of ovarian PMF reserve via inhibition of CHEK2. However, potent inhibition of CHEK1 resulted in cytotoxicity and apoptosis of ovarian somatic cells limiting utility of AZD7762 as an ovario-protective agent. Inhibition of CHEK2 by AZD7762 was also reported to increase bone remodeling, indicating that better CHEK1/2 inhibitors may be used to treat cancer and prevent bone loss during bone metastasis (*85*). Genetic and pharmacological inhibition of CHEK2 in lymphoid and myeloid cell lines reduced PARPi-induced hematologic toxicity, further demonstrating that specific CHEK2 inhibition may be used to alleviate cancer treatment–induced toxicities (*86*). Unfortunately, unpredictable cardiotoxicity of AZD7762 in combination with gemcitabine led to termination of clinical trials (*87*), but CHEK1/2 kinases continue to be considered as therapeutic targets (*52*). *Chek2^−/−^* mice are viable and fertile, and do not develop tumors without other oncogenic mutations. This suggests that transient inhibition of CHEK2 would be an efficient strategy to preserve PMF reserve and ovarian endocrine function and potentially alleviate other adverse effects if inhibitors with higher selectivity toward CHEK2 than CHEK1 can be identified. While this study reveals that CHEK2 inhibition may protect PMF reserve from multiple chemotherapies, it has certain limitations. For example, it remains unknown whether the genome of preserved drug-treated oocytes would be intact. Analysis of DSB repair in drug-treated oocytes revealed reduced numbers of drug-induced DSBs after AZD7762 inhibition, suggesting an ongoing DNA repair at the end of explant culture. Our previous work showed that extensive DNA damage caused by meiotic recombination failure is efficiently repaired in *Chek2^−/−^* oocytes resulting in healthy offspring (*19*). Moreover, offspring born from radiation-damaged oocytes surviving in *TAp63^−/−^* females were healthy and whole genome sequencing did not detect increased mutation rates, although the number of sequenced animals was limited (*38*). It is important to note that primordial oocytes are arrested at meiotic prophase I (G2), with all four chromatids in close juxtaposition, during which error-free HR is the predominant repair pathway. This fact potentially diminishes the risk that chemotherapy-induced DSBs will result in mutations. Additionally, the likelihood of any mutation being transmitted to offspring is limited by the biological processes of meiosis whereby only one of the four parental chromatids is incorporated into the fertilized egg. Nonetheless, additional comprehensive studies utilizing whole genome sequencing will be necessary to fully evaluate the safety of targeting CHEK2 in oocytes destined for reproduction. CHEK2 inhibition may prove more suitable for patients who prefer not to undergo ovary removal and cryopreservation for reproductive purposes, but desire to reduce the risk of premature menopause and other health complications associated with reproductive aging. Although the mouse model shows no adverse consequences of CHEK2 deficiency, an important consideration is that transient inhibition of CHEK1, CHEK2, or other checkpoint proteins can lead to temporary weakening of the mechanisms guarding genomic integrity. Germline mutations in *CHEK2* have been found in patients with breast, colon, and prostate cancers (*88*). Therefore, further studies are warranted to evaluate the effectiveness and safety of transient CHEK2 inhibition in animal models with a genetic predisposition to cancer. In summary, our findings highlight the critical role of CHEK2 in regulating the depletion of ovarian follicle reserve caused by radiation and chemotherapy. Targeting CHEK2 with selective inhibitors could be a potential strategy to protect ovaries during cancer treatments and mitigate ovarian aging in cancer survivors.

## MATERIALS AND METHODS

### Animals

All procedures used in this study were approved by the Institutional Animal Care & Use Committee (IACUC) at the Jackson Laboratory. C57BL/6J (JAX stock#000664), Tg(Pou5f1-EGFP)2Mnn/J (JAX stock#004654), and *Trp53^tm1Tyj/J^* (JAX stock#002101) were obtained from the Jackson Laboratory. *Chek2^tm1b(EUCOMM)Hmgu^* mice were obtained from KOMP program at JAX. The *Trp63*^S621A^ mutant line was generated using CRISPR-Cas9–mediated genome editing as described in fig. S2. For radiation experiments, 7-day-old females were irradiated using a Cesium-137 gamma irradiator. They were exposed to a total dose of 0.5 or 3 Gy administered at a rate of (~170 rad/min). Ovaries were collected for protein extracts for Western blot analysis or fixed in 4% PFA for immunostaining. For chemotherapy drug treatments, 7-day-old females received a single i.p. injection of saline, CDDP (5 mg/kg) (Millipore Sigma), or CTX (150 mg/kg) (Millipore Sigma). Doses were chosen based on previously published studies (*64*). Mouse weights were recorded 1 and 2 weeks after injection. Both ovaries from each female were collected 2 weeks after injection, fixed in Bouin’s solution for histological analyses.

### Follicle quantification

Bouin’s fixed ovaries were embedded in paraffin and cut into 5 μm serial sections. Sections were stained with Periodic acid-Schiff (PAS) and follicle number was evaluated in every fifth section. Follicle stages were determined using standard methods. Briefly, PMFs were surrounded by a layer of squamous pre-granulosa cells; primary follicles had a single layer of cuboidal granulosa cells; secondary follicles had more than one granulosa cell layer but lacked antrum; antral follicles had visible antrum. Final average follicle count per ovary per female is represented as the sum of follicles in every fifth section multiplied by a correction factor of 5 (*89*). Percentage survival was calculated by normalizing oocyte counts per treated ovary to the average oocyte number of the vehicle control group for the same genotype [i.e., % survival = (WT Drug/mean WT Veh)*100%].

### Organ culture and drug ex vivo treatments

Ovaries collected from 7-day-old pups were cultured on polycarbonate membrane (Whatman Nucleopore Polycarbonate membrane) in MEM (Gibco) supplemented with 10% fetal bovine serum (FBS, Seradigm), 25 mM Hepes (Lonza), and 1× Pen/Strep (Gibco). Ovarian explants were incubated at 37°C, 5% CO_2_, and atmospheric O_2_. Chemotherapy drugs and inhibitors were purchased in powder form and reconstituted following the manufacturer’s recommendations: CDDP (Selleckchem), MAFO (CTX-analog, US Biological), DOX (Selleckchem), ETO (Merck Millipore), AZD7762 (IC50 CHEK2 10 nM and CHEK1 5 nM) (Selleckchem), CCT241533 (IC50 CHEK2 3 nM and CHEK1 245 nM) (TOCRIS), LY2606368 (IC50 CHEK2 8 nM and CHEK1 < 1 nM) (Selleckchem), PF477736 [IC50 CHEK2 47 nM(Ki) and CHEK1 0.49 nM(Ki)] (TOCRIS). Stock solutions for all compounds were prepared in dimethyl sulfoxide (DMSO) except for CDDP, which was dissolved in dimethylformamide (DMF) (as recommended by the supplier). DMSO and DMF were used as vehicle controls and their resulting concentration in culture media was less than 0.1%. We used MAFO in ex vivo culture to imitate the in vivo activity of CTX, which is metabolized by the liver to generate metabolites with alkylating properties (*28*, *90*). During drug treatments, ovaries were cultured with drugs for 2 days and then cultured in drug-free medium for 5 days before further experimentation (7 days total). The culture medium was changed every 2 days. During inhibitor treatments, ovaries were cultured with inhibitors for 2 hours before irradiation or drug treatment. After radiation (0.5 Gy), medium was changed to inhibitor-free ~24 hours after radiation. When ovaries were cultured with drugs, replacement to normal medium (drug and inhibitor-free) occurred at 48 hours after treatment start. Ovaries were cultured for a total of 7 days for oocyte survival analyses or for 3 to 24 hours for protein analyses. Culture experiments were repeated two to three times each with ≥1 ovary per genotype (depending on litter size and genotype availability). Typically, ovaries from females of the same genotype dissected at the same time were randomly distributed between vehicle and treatment samples to reduce the number of experimental animals. In some cases, one ovary per female was used for vehicle control and the other one for treatment. Drug and inhibitor concentrations used were as follows: CDDP: 0.1 μg/ml (0.3 μM), 0.25 μg/ml (0.8 μM), and 0.5 μg/ml (1.6 μM); MAFO: 0.1 μg/ml (0.4 μM), 0.5 μg/ml (1.2 μM), and 1 μg/ml (2.4 μM); DOX: 25 ng/ml (43.1 nM), 50 ng/ml (86.2 nM), and 100 ng/ml (172.5 nM); ETO: 50 ng/ml (84.5 nM), 100 ng/ml (170 nM), and 500 ng/ml (849.5 nM); AZD7762 (0.1, 1, and 10 μM), CCT241533 (0.05, 0.5, and 5 μM), LY2606368 (0.1, 1, and 10 μM), and PF477736 (0.5, 5, and 50 μM).

### Immunohistochemistry and TUNEL staining

Slides with 5-μm ovarian sections were processed using standard methods. Briefly, sections were deparaffinized and re-dehydrated before antigen retrieval using sodium citrate buffer (10 mM sodium citrate and 0.05% Tween 20, pH 6.0). Sections were permeabilized in 0.2% Triton X-100 in phosphate-buffered saline (PBS) and blocked with 10% goat serum for 1 hour, before incubation with primary antibodies at 4°C overnight. Secondary antibodies were applied for 1 hour and slides were mounted with VectaShield (Vector Laboratories) with DAPI (4′,6-diamidino-2-phenylindole) or after Hoechst 33342 (Biotechne) staining. Primary antibodies used in this study were mouse anti-p63 (4A4, Biocare Medical, CM163A), rabbit anti-pCHEK2(T68) (Bioworld Technology, BS4043: validated in *Chek2^−/−^* tissue), rabbit anti-phospho-p53(S15) (Cell Signaling #9284), rabbit anti-DDX4 (Abcam, ab13840), rabbit anti-FOXL2 (Abcam ab275153), mouse anti-γ-H2AX (Millipore, 05–636), rabbit anti-RAD51 (Abcam, ab176458), and rabbit anti-53BP1(Novus NB100–304). Secondary antibodies used were Alexa Fluor (Invitrogen). Immunostaining for phospho-p53(S15) was performed with Starr Trek reagent (Biocare Medical). Antibody co-immunostaining with TUNEL for apoptotic cells (DeadEnd Fluorometric system, Promega, G3250) was performed by standard immunofluorescence protocol followed by TUNEL staining according to the manufacturer’s protocol. After completion of TUNEL staining, sections were incubated again with secondary antibodies.

### Whole mount staining of ovaries

Whole mount immunostaining of cultured ovaries was performed as described in (*91*). Briefly, explanted ovaries attached to the polycarbonate membrane were fixed in 2% PFA at 4°C overnight. Fixed ovaries were washed with 70% ethanol overnight and placed in PBS for at least 4 hours before immunostaining. Ovaries were permeabilized in solution containing 0.2% polyvinyl alcohol (PVA), 0.1% NaBH_4_, and 1.5% Triton X-100 in PBS for 4 hours, and incubated in blocking solution [10% goat serum, 3% bovine serum albumin, 0.15% glycine, pH 7.4, 0.1% Triton X-100, 0.2% sodium azide, penicillin (100 U/ml), and streptomycin (100 ng/ml), in PBS] overnight. Primary antibody incubation was performed at room temperature with gentle rocking for 2 to 4 days. Primary antibodies used were mouse anti-p63 (4A4, Biocare Medical, CM163A) and rabbit anti-DDX4 (Abcam, ab13840). Ovaries were washed with wash solution (0.2% PVA and 0.15% Triton X-100, in PBS) for 1 to 2 days with buffer changes (2× daily). Ovaries were next incubated with Alexa Fluor secondary antibodies for 2 to 3 days followed by washing for 1 to 2 days with buffer changes.

### Optical clearing, imaging, and quantification of oocytes

Optical clearing of immunostained ovaries was performed as described in (*91*). Briefly, ovaries were cleared using ScaleS4(0) [40% d-(−)-sorbitol (w/v), 4 M urea, 10% glycerol, and 20% DMSO, in PBS]. ScaleS4(0) solution was refreshed twice daily until tissues became cleared (2 to 3 days). The membranes with ovaries were mounted using CoverWell Incubation Chamber (Research Products International) and imaged using a Leica DM550 microscope. Images were collected as Z-stacks (5 μm) and used to generate maximum projection image in LAS X software. Oocytes were counted using markers DDX4 (cytoplasmic staining) and p63 (nuclear staining). Small oocytes with a diameter less than 30 μm (delineated by DDX4 staining) were categorized as primordial oocytes and those larger than 30 μm were categorized as growing oocytes (corresponding to growing follicles including primary and secondary follicles). Percentage survival was calculated by normalizing oocyte counts per ovary to the average oocyte number of the vehicle control group for the same genotype [i.e., % survival = (WT Drug/mean WT Veh)*100%].

### Immunoblots

Protein extracts were prepared with radioimmunoprecipitation assay buffer supplemented with protease and phosphatase inhibitors (Sigma-Aldrich) using a minipestle or Bioruptor Pico (Diagenode). Proteins were resolved in 10% acrylamide gel and transferred to nitrocellulose membranes. Primary antibodies used in this study were rabbit anti-p63 (Cell Signaling Technology, 13109), rabbit anti-total p53 (Leica, CM5P), mouse anti-γ-H2AX (Millipore, 05-636), rabbit anti-pCHEK1 (S317) (Cell Signaling #12302S), rabbit anti-DDX4 (Abcam, ab13840), and mouse anti-ACTB (GeneTex, GT5512). After incubation with HRP secondary antibody, signals were detected using Luminata Forte/Crescendo Western HRP substrate (Millipore). For probing with multiple antibodies, membranes were stripped by using Western blot stripping buffer Restore (Thermo Fisher Scientific).

### Statistics

Statistical analysis was performed using PRISM 9.0 (GraphPad Software). To analyze the difference between more than two independent groups (e.g., vehicle versus drug doses) statistical analysis was performed by one-way analysis of variance (ANOVA) and the significance was determined by Bonferroni multiple-comparison test or Kruskal-Wallis for nonparametric data with Dunn’s multiple-comparison test. For pairwise comparisons (e.g., wild type versus mutant) significance was determined by *t* test or Mann-Whitney nonparametric test. Values of *P* < 0.05 were considered statistically significant. Data are presented as means ± SEM.*P < 0.05; **P < 0.01; ***P < 0.001; ****P < 0.0001; n.s., nonsignificant.
