# Supplementary material for: CHEK2 signaling is the key regulator of oocyte survival after chemotherapy
Source: Sci Adv. 2023 Oct 20;9(42):eadg0898. doi: 10.1126/sciadv.adg0898 (PMC10588956; doi:10.1126/sciadv.adg0898)
Supplement: Supplementary file 1 — Figs. S1 and S2 [file sciadv.adg0898_sm.pdf]

Science Advances
AAAS

Supplementary Materials for

CHEK2 signaling is the key regulator of oocyte survival after chemotherapy

Chihiro Emori *et al.*,

Corresponding author: Ewelina Bolcun-Filas, Ewelina.Bolcun-Filas@jax.org

Sci. Adv. **9**, eadg0898 (2023)
DOI: 10.1126/sciadv.adg0898

This PDF file includes:

Figs. S1 and S2

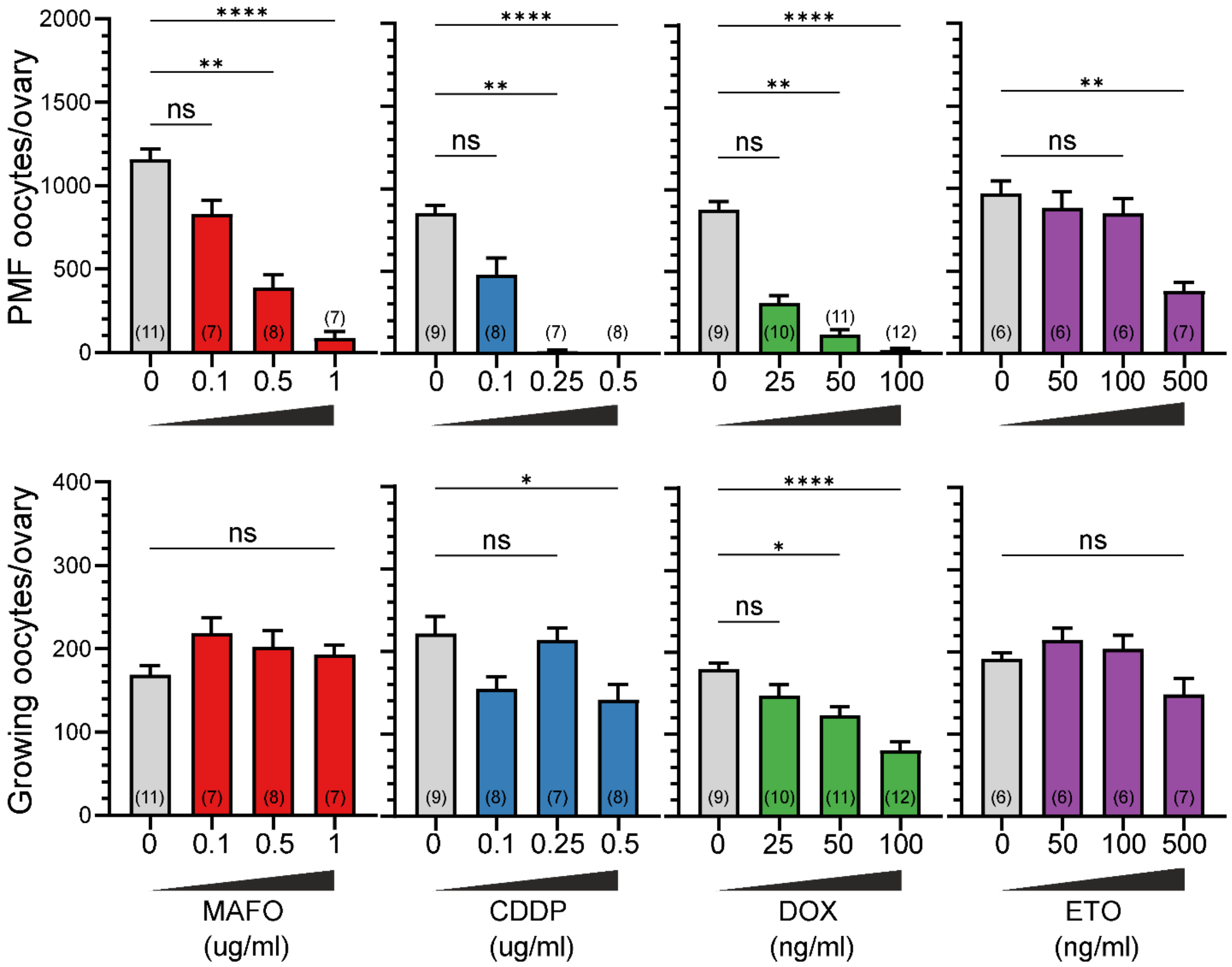

Fig. S1.

Ovotoxicity of alkylating agents (MAFO and CDDP) and topoisomerase II poisons (DOX and ETO) is dose dependent. Ovaries from wildtype Tg(Pou5f1-EGFP)2Mnn/J females were treated for 48hrs with MAFO (0.1, 0.5, 1µg/ml), CDDP (0.1, 0.25, 0.5µg/ml), DOX (25, 50, 100ng/ml) or ETO (50, 100, 500ng/ml). Primordial and growing oocytes were counted in ovaries harvested after 7-day organ culture. Primordial oocytes are found in PMFs while larger growing oocytes are found in primary and secondary follicles present in 2-weeks-old ovaries. Sample number (N); number of ovaries per group. Data are expressed as mean ± SEM; *p<0.05, **p<0.01, ***p<0.001, ****p<0.0001 (one-way ANOVA, Kruskal–Wallis with Dunn's multiple comparison test for nonparametric data).

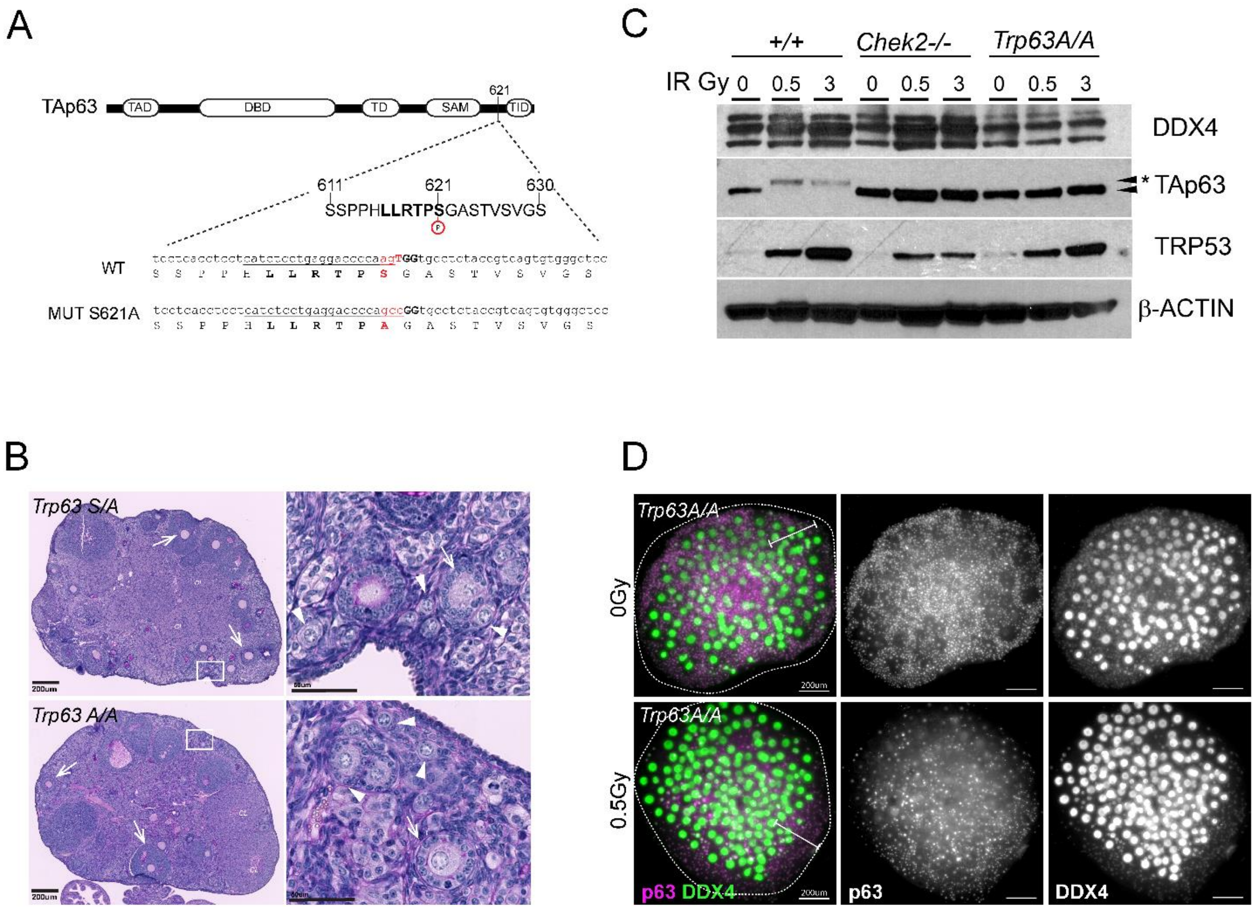

Fig. S2.

Mutation of 621 Serine to Alanine abolishes TAp63 phosphorylation and leads to its inactivation. (**A**) Schematic view TAp63 protein domain structure and localization of 621 Serine mutated by CRISPR-Cas9 editing. PAM site is shown in bold and sgRNA sequence is underlined. (**B**) *Trp63A/A* females have normal ovarian histology with abundant PMF (arrowheads) and growing follicles (arrows). (**C**) Ovarian protein extracts were collected 3hrs after radiation with 0.5 and 3Gy from wildtype *Chek2-/-* and *Trp63A/A* ovaries and were analyzed by western blot. TAp63 mobility shift is observed in wildtype ovaries (asterisk) but not in CHEK2 deficient or *Trp63A/A* mutant. p53 expression is detected following radiation even in the absence if CHEK2 although at lower levels than in the wildtype, indicating that CHEK1 or other kinases can still activate p53 in the absence of CHEK2. (D) PMF survive in *Trp63A/A* ovaries after low dose of radiation (0.5Gy). White bars indicate regions where PMF are typically found in cultured ovaries. Scale bar 200μm.
